# Serum Dehydroepiandrosterone Sulphate Concentration Is Not a Predictive Factor in IVF Outcomes before the First Cycle of GnRH Agonist Administration in Women with Normal Ovarian Reserve

**DOI:** 10.1371/journal.pone.0118570

**Published:** 2015-03-04

**Authors:** Michał Kunicki, Krzysztof Łukaszuk, Grzegorz Jakiel, Joanna Liss

**Affiliations:** 1 INVICTA Fertility and Reproductive Center, Warszawa, Poland; 2 Department of Obstetrics and Gynaecological Nursing, Faculty of Health Sciences, Medical University of Gdansk, Gdańsk, Poland; 3 Department of Obstetrics and Gynaecology, Faculty of Medical Sciences, University of Varmia and Masuria, Olsztyn, Poland; 4 NVICTA Fertility and Reproductive Center, Gdańsk, Poland; 5 Department of Obstetrics and Gynecology, Medical Center of Postgraduate Education, Warsaw, Poland; Women’s Hospital, School of Medicine, Zhejiang University, China. 310006, CHINA

## Abstract

**Objective:**

The aim of our study was to determine whether serum dehydroepiandrosterone sulphate (DHEAS) concentration and the models incorporating it could help clinicians to predict IVF outcomes in women with normal ovarian reserve undergoing their first long protocol.

**Study Design:**

We performed a retrospective analysis of 459 women undergoing cycles of intracytoplasmic sperm injection (ICSI) for the first time in a long GnRH agonist protocol.

**Results:**

Embryo transfer was performed in 407 women (88.7%). The fertilisation rate was 78.6%. The clinical pregnancy rate was 44.8% per started cycle and 50.6% per embryo transfer. Our univariate model revealed that the best predictors of clinical pregnancy were the number of mature oocytes, the number of embryos transferred and the number of good quality embryos, account for the clinical parameters that reflect ovarian reserve the best being AMH level and AFC. DHEAS did not predict clinical pregnancy (OR 1.001, 95% CI, 0.999–1.004). After adjusting for the number of embryos transferred and class of embryos in a multivariate model, the best predictors were age (OR 0.918, 95% CI, 0.867–0.972) and AFC (OR 1.022, 95% CI, 0.992–1.053). Serum DHEAS levels were positively correlated with AFC (r = 0.098, P<0.039) and testosterone levels (r = 0.371, P<0.001), as well as the number of mature oocytes (r = 0.109, P<0.019); serum DHEAS levels were negatively correlated with age (r = -0.220, P<0.001), follicle-stimulating hormone (FSH), (r = -0.116, P<0.015) and sex hormone-binding globulin (SHBG), (r = -0.193, P<0.001).

**Conclusions:**

DHEAS concentration (in addition to the known factors of ovarian reserve) does not predict clinical pregnancy in women with normal ovarian reserve who are undergoing ICSI.

## Introduction

Numerous factors affect IVF outcomes [1]. The most important factors are the patient’s age and her AMH level [2,3,4]. In clinical practice, it is of utmost importance for doctors and patients to be able to predict IVF outcomes before the first cycle. Single parameters, such as AMH, have limited value in predicting who can actually conceive [5]. The assessment of a women’s age, combined with her AMH level, appear to be the gold standard [6]. However, models combining the two aforementioned parameters appear to have only moderate value in predicting clinical pregnancy and live birth [7]. Serum dehydroepiandrosterone sulphate (DHEAS) concentration could improve IVF results for women with low ovarian reserve in the first antagonist cycle [8]. Based on the aforementioned data, the aim of our study was to investigate whether DHEAS concentration and the models incorporating it could help clinicians to predict IVF outcomes in women with normal ovarian reserve who are undergoing their first long protocol.

## Material and Methods

We searched our database for cases from 2005 to 2010. The following inclusion criteria were applied:

- women who were undergoing their first long GnRH agonist protocol,
- women with measured DHEAS concentrations,
- women with only fresh blastocyst transfers,
- women who had AMH measured with DSL kits,
- women with a normal uterine cavity,
- women who had ICSI cycles,
- women who were not using any medicines that could interfere with their basal hormonal status, and
- women with no severe male factor (i.e., sperm count less than 10^6^ ml).

We applied the following exclusion criteria:

- oocyte donor program participating and frozen-thawed sperm,
- prenatal genetic diagnosis and screening,
- cleavage embryo transfer,
- androgen supplementation within six months before enrolling in the study, and
- the presence of polycystic ovarian syndrome.

As described in detail previously [9], the long GnRH agonist protocol used in our centre was based on oral contraceptive treatment with ovulastan (Polfa, Pabianice, Poland), started between days 1 and 5 of a spontaneous or induced menstrual cycle. Beginning on treatment day 16 or 17, triptorelin (Ferring, Germany) was administered every two days for 14 days. Gonadotropin treatment was initiated 14 days later. Human menopausal gonadotropins (HMGs) (Menopur, Ferring, Germany) were initiated for ovarian controlled hyperstimulation in a step-down fashion and were continued until at least three follicles > 17 mm were visualised.

Follicular growth was monitored on treatment day 8 with an ultrasonographic scan and serum oestradiol measurement. Ovulation was then induced by administering 5,000 IU of hCG (Choragon, Ferring, Germany). Oocyte retrieval was performed by transvaginal aspiration 36 hours after hCG administration.

Embryo transfer occurred during the blastocyst stage on day 5. The number of transferred embryos was determined by the guidelines of the American Society for Reproductive Medicine [10]. The spare embryos were frozen, and their outcomes are not discussed in the manuscript. The grading of embryos was performed according to Gardner’s classification [11]. Embryos graded as 2 BB or better was classified as “good quality embryos.” Continuous luteal support was started after oocyte retrieval. All of the patients were administered a natural micronised progesterone supplement (Luteina, Adamed, Czosnów, Poland), at 600 mg/d, plus 6 mg of micronised 17 beta-oestradiol (Estrofem, Novo Nordisk, Denmark), which was administered in three daily oral doses. Serum HCG was measured 16 days after oocyte retrieval. Clinical pregnancy was defined by the presence of a gestational sac on ultrasonography 5 to 6 weeks after embryo transfer.

### Ethics statement

All patients provided written informed consent, and this investigation was approved by the Institutional Review Board at Komisja Bioetyczna przy Okręgowej Izbie Lekarskiej w Gdańsku, trial registration number KB-17/14.

### Hormone assays

The serum levels of prolactin (PRL), follicle-stimulating hormone (FSH), luteinising hormone (LH), oestradiol (E_2_), progesterone (P), testosterone (T), and sex hormone-binding globulin (SHBG) were measured with an electrochemiluminescence immunometric assay using commercial kits (Cobas 6000, e601). We collected venous blood samples between the first and third days of the menstrual cycle, before stimulation. The intra- and inter-assay coefficients of variation (CV), respectively, were 3% and 3.3% for PRL, 7.4% and 4.7% for E_2_, 2.5% and 2.3% for FSH, 2.2% and 2.4% for LH, 4.9% and 5% for T, 3% and 4.7% for SHBG, and 3.3% and 3.2% for DHEAS. Serum inhibin B and AMH were measured by enzyme-linked immunosorbent assay (ELISA) (Diagnostic Systems Laboratories, Webster, TX, USA). The assay’s limit of detection was 0.06 ng/ml for AMH and 7 pg/ml for inhibin B. The intra- and inter-assay coefficients of variation for inhibin B and AMH were <10%. The reference intervals in the follicular phase were as follows: serum, PRL 90–540 mU/L; FSH, 2.4–9.3 U/L; LH, 2.0–12.0 U/L; E_2_, 12.5–166 pg/ml; T, 0.1–0.9 ng/ml; SHBG, 20.0–118 ng/ml; and DHEAS, 98.8–340 μg/dl.

### Statistics

Baseline data are expressed as median values and first—third quartile (continuous data) or as numbers and percentages of patients (discrete data). The distribution of data was analysed using the Kolmogorov-Smirnov test. None of the variables were normally distributed. The baseline patient characteristics and differences between pregnant and non-pregnant women were compared using the Mann-Whitney *U*-test. For the prediction of live births, logistic regression analyses were used. The results are expressed as odds ratios (ORs), with 95% confidence intervals (CIs). The Wald test multivariate logistic regression was also used (adjusted by the number of embryos transferred). Accuracy, sensitivity, specificity, and positive (PPN) and negative predicative values (NPV) were calculated for this analysis. Spearman’s correlation coefficient was used to assess the relationship between DHEAS and other variables. A p value of <0.05 was considered to be statistically significant. All analyses were performed using R version 2.1.3.0.

## Results

In our database, we searched the records of 5136 women participating in IVF programs at our centre. Finally, in accordance with the inclusion and exclusion criteria, 459 women were included in the study. The records were from women undergoing their first long protocol. The median duration of infertility was 4 years. The causes of infertility were anovulation (17.4%), tubal factors (14.0%), male infertility (25.7%), endometriosis (1.3%), unexplained (6.1%) and mixed (35.5%). There were no significant differences in the rates of any causes between the women who conceived and those who did not (p = 0.431).

Embryo transfer was performed in 407 women (88.7%). Fifty-two women had the cycle cancelled due to a lack of oocytes on pick-up, a lack of fertilisation, failure to respond to gonadotropin or excessive response. The fertilisation rate was 78.6%. The implantation rate was defined as the number of sacs with heartbeats divided by the number of embryos transferred, and this rate was 23.7%.

The clinical pregnancy rate was 44.8% per started cycle and 50.6% per embryo transfer. The live birth rate was 30.3% per started cycle and 34.1% per embryo transfer. There were 108 live single deliveries, 9 twin deliveries and 1 triplet delivery.

The baseline characteristics of the study population are shown in table 1. There were no significant differences in DHEAS between the women who conceived and those who did not (p = 0.868). Moreover, no differences in PRL, PRL after metoclopramide, FSH, LH, E_2_, T, or SHBG were observed between the women who conceived and those who did not. A comparison of their baseline characteristics showed significant differences in age, AMH, AFC, number of cumuli and number of mature oocytes. Women who did not conceive required more gonadotropins (p = 0.026). Additionally, the women who conceived had more good quality embryos transferred than women who did not (p<0.001).

**Table 1:** The baseline characteristics of the study population and differences between pregnant and non-pregnant women.

	All N = 459	Pregnant N = 206	Nonpregnant N = 253	P
Age (y)	32 (29–35)	31.5 (29–34)	33 (30–36)	<0.001
Total gonadotropin dose(75-IU)	20 (17–26)	20 (16–24)	21 (17–27)	0.026
Duration of stimulation (days)	9 (8–10)	9 (8–10)	9 (8–10)	0.384
AMH ng/ml	3.7 (2.2–6)	4 (2.5–6.5)	3.4 (1.8–5.5)	0.005
Inhibin B pg/ml	56 (32.8–81.1)	56.4 (30.1–77.8)	54.6 (33–85)	0.673
AFC	12 (7–17)	13 (9–18)	10 (6–15)	<0.001
Number of cumulus	11 (6–16)	12.5 (9–17)	9 (5–14)	<0.001
Number of mature oocytes	7 (4–11)	8 (6–12)	6 (2–9)	<0.001
Fertilisation rate	78.6 (57.1–96.3)	80.0 (62.5–91.7)	75.0 (50.0–100)	0.307
Number of embryos transferred				<0.001
0	52 (11.3%)	-	52 (20.6%)	
1	54 (12.8%)	13 (6.3%)	41 (16.2%)	
2	323 (70.4%)	179 (86.9%)	144 (59.9%)	
3	30 (6.5%)	14 (6.8%)	16 (6.3%)	
Good quality of embryos	207 (of 407) (50.8%)	130 (63.1%)	77 (38.1%)	<0.001
LH (IU/L)	5.8 (4.5–7.4)	6 (4.6–7.6)	5.8 (4.4–7.3)	0.198
FSH(IU/L)	7.3 (5.9–8.8)	7.1 (5.8–8.8)	7.4 (6–8.9)	0.738
E_2_ (pg/ml)	40 (29.9–57)	40.3 (29–57.5)	40 (30–57)	0.872
T (ng/ml)	1.1 (0.8–1.5)	1.1 (0.8–1.6)	1.1 (0.7–1.4)	0.116
PRL 0′ (mU/L)	310 (212–479)	332.5 (191–479)	293 (218.5–475)	0.875
PRL 60′ (mU/L)	4130.5 (2734–5109)	3957(2785.5–5045.5)	4346 (2562–5222)	0.787
DHEA-S (μg/dl.)	197 (146–256)	201.5 (143–252)	195 (146–259)	0.868
SHBG (ng/ml)	60 (43.7–83)	61 (45–91)	59 (43.4–77)	0.268
BMI (kg/m^2^)	21.9 (20.1–24.2)	21.4 (19.7–24.1)	22.3 (20.3–24.3)	0.108
Durationof infertility(y)	4 (1.4–4)	4 (1–4)	4 (3–4)	0.159

All data are the median (Q1–Q3). The p-values are from the Mann-Whitney *U*-test

We did not find any significant difference in serum DHEAS concentrations between the women who had good quality embryos transferred and those who did not (204 vs 200 μg/dl).

Table 2 presents the univariate regression analysis of variables studied for the prediction of clinical pregnancy. The best predictors of pregnancy were the number of embryos transferred and the quality of the embryos. From the clinical point of view, AFC was the best single predictor of clinical pregnancy. The second-best predictive factors were AMH and age. Serum DHEAS did not predict clinical pregnancy (OR 1.001; 95%, CI, 0.999–1.004).

**Table 2:** Univariate regression analysis of the variables predicting clinical pregnancy.

	OR	95%CI	p
Age (y)	0.903	0.858–0.950	<0.001
Total gonadotropin dose	0.972	0.940–1.005	0.095
Duration of stimulation (days)	1.070	0.954–1.201	0.248
AMH (ng/ml)	1.074	1.011–1.141	0.021
Inhibin B (pg/ml)	0.999	0.994–1.005	0.842
AFC	1.060	1.029–1.092	<0.001
Number of cumulus	1.076	1.042–1.111	<0.001
Number of mature oocytes	1.084	1.038–1.132	<0.001
Number of embryos transferred	4.040	2.554–6.338	<0.001
Good quality embryos	2.777	1.860–4.146	<0.001
LH (IU/L)	1.023	0.963–1.086	0.457
FSH (IU/L)	0.997	0.991–1.004	0.449
E_2_ (pg/ml)	0.997	0.991–1.003	0.335
T (ng/ml)	1.238	0.883–1.735	0.216
PRL 0′ (mU/L)	1.000	0.998–1.002	0.893
PRL 60′ (mU/L)	1.000	1–1	0.773
DHEAS (μg/dl.)	1.001	0.999–1.004	0.271
SHBG (ng/ml)	0.181	0.998–1.010	0.181
BMI (kg/m^2^)	1.005	0.998–1.013	0.150
Duration of infertility (y)	1.104	0.749–1.626	0.617

Table 3 presents the multivariate model for clinical pregnancy combining age, AFC, AMH and FSH as the predictors of clinical pregnancy.

**Table 3:** Multivariate logistic regression analysis of age, AFC, AMH and FSH as the variables predicting clinical pregnancy, adjusted by the number of transferred embryos and class of embryos.

	OR	95% CI	p	AUC	95% AUC	Accuracy	Sensitivity	Specificity	PPN	NPV
age	0.911	0.853-	0.005	0.69	0.636-	66.5	72.2	60.3	66.1	66.9
		0.972			0.753					
AFC	1.014	0.975-	0.491							
		1.053								
AMH	1.018	0.938	0.664							
		1.106-								
FSH	1.038	0.953-	0.393							
		1.130								

The accuracy, sensitivity, specificity, and positive (PPN) and negative predicative values (NPV) are shown as percentages.

Table 4 presents the multivariate model for clinical pregnancy combining age and AFC as the predictors of clinical pregnancy.

**Table 4:** Multivariate logistic regression analysis of age and AFC as the variables predicting clinical pregnancy, adjusted by the number and class of transferred embryos.

	OR	95% CI	p	AUC	95% AUC	Accuracy	Sensitivity	Specificity	PPN	NPV
age	0.918	0.867-	0.003	0.69	0.639-	65.7	87.7	42.69	61.4	77.1
		0.972			0.743					
AFC	1.022	0.992-	0.158	0.69	0.639-					
		1.053			0.743					

The accuracy, sensitivity, specificity, and positive (PPN) and negative predicative values (NPV) are shown as percentages

The serum DHEAS level was positively correlated with AFC (r = 0.098, P<0.039), number of mature oocytes (r = 0.109, P<0.019), and testosterone (r = 0.371, P<0.001) and negatively correlated with age (r = -0.220, P<0.001), FSH (r = -0.116, P<0.015) and SHBG (r = -0.193, P<0.001).

## Discussion

The main purpose of our study was to investigate whether DHEAS concentration is a useful tool in predicting clinical pregnancy, in addition to the known factors of ovarian reserve. In reproductive medicine, the woman’s age and AMH level are the main parameters used as independent predictors of IVF success [1]. Separately, age and AMH have limited value in predicting pregnancy and live birth after IVF [12–14]. Numerous alternative models that predict treatment success incorporate the number of oocytes retrieved, fertilisation rate, and the number and quality of transferred embryos [15–17]. The number of fertilised oocytes and the number of retrieved oocytes have the highest prognostic value [18]. AMH and AFC have established roles in predicting the number of retrieved oocytes before stimulation [19].

The rationale for this study was to improve the prediction of clinical pregnancy, thereby improving pregnancy counselling. Most IVF counselling studies have focused on women with poor ovarian reserve (POR), as well as determining the role of DHEA supplementation in improving oocyte and embryo quality [3,18–22]. Our work was motivated by the study of Alebić et al. [8,23], who presented data on the predictive value of DHEAS in women with poor ovarian reserve. In that study, 129 women with low AMH participated in a short antagonist protocol, and DHEAS appeared to be predictive for clinical pregnancy in younger women (younger than 37.5 years of age) with low AMH. Moreover, the predictive power of combining age and DHEAS was significantly higher compared to that of age alone [8]. In a second study, the same group investigated the ability of DHEAS to predict live birth before starting the first gonadotropin-releasing hormone antagonist ovarian stimulation for IVF/ICSI in women younger than 37 years with low serum AMH. The authors found that in women with DHEAS concentrations >5.4 μmol/l, the live birth rate was 5-fold greater compared to women with DHEAS concentrations <5.4 μmol/l [23]. Guo et al. investigated whether DHEAS or basal testosterone concentration could be an effective predictor of POR (defined by the Bologna criteria). The authors found that DHEAS concentration was not significantly different between poor and normal responders or between pregnant and nonpregnant women. Basal testosterone, unlike DHEAS, was predictive of POR, but its ability as a single predictor was limited [24].

In our study, we included women with normal AMH levels who were undergoing their first agonist cycle. Different studies have published different cut-off values for AMH, below which the chances for pregnancy and/or live birth are reduced [14,19]. La Marca et al. and Nelson et al. independently demonstrated that AMH cut-off values between 0.7 and 0.75 ng/ml predict poor ovarian response [14,25]. According to these data, we only included women with AMH levels >0.7 ng/ ml. Additionally, in this study we included only women who underwent blastocyst transfers. In our centre, 3-day embryo transfers are very rarely performed, mainly in women with low ovarian reserves and those older than 40 years old.

After comparing the parameters of women according to their pregnancy status, we found that the women who conceived were younger, with higher AFCs and AMH levels and a greater number of retrieved oocytes. These women had also more good quality embryos transferred into the uterus. We did not find any between-group differences in terms of the DHEAS concentration, although a slightly higher level was observed in the women with clinical pregnancy (201.5 vs 194.5 μg/ml). Our univariate model revealed that the best predictors of clinical pregnancy were the number of mature oocytes, the number of embryos transferred and the number of good quality embryos, account for the clinical parameters that reflect ovarian reserve the best being AMH level and AFC. Unfortunately, DHEAS was not a predictor for clinical pregnancy. Thus, we could not incorporate DHEAS into a model including age and AMH (as in the study of Alebić et al.) to create a complex model. In further analysis, we tested models that incorporated known ovarian reserve markers, such as FSH, AFC, and AMH, adjusting the models for number of embryos transferred and class of embryos. We tested also other models in which AMH was excluded from further analysis. After adjusting for the number of embryos transferred and class of embryos in a multivariate model, the best predictors were age and AFC (table 4). Thus, we presented results in which DHEAS had limited value in predicting clinical pregnancy. It is worth noting that our data were in agreement with those presented by the Fratarelli and Gerber study, in which only testosterone and androstenedione serum concentrations, which are ovarian in origin, were correlated with the number of oocytes retrieved and the number of mature oocytes, but they did not predict pregnancy [26].

Our study has some limitations. First, as a retrospective trial, it was subject to certain biases. Second, we included only the women who were undergoing a long protocol and blastocyst transfers, which we discussed previously in this manuscript. It would be appropriate to test women with different protocols in future studies. The dosage of gonadotropins was flexible, according to the patient age, basal AFC, and basal AMH level. Third, we chose 0.7 ng/ml AMH as the concentration level below which women were classified as having low ovarian reserve. As stated above, different studies have established different AMH cut-offs, such as 0.4 ng/ml [27,28]. We also tested different AMH concentrations (e.g., AMH>25th percentile), and DHEAS still was not predictive (data not shown). Note that we measured AMH using the DSL assay; therefore, our data do not include the women for whom the AMH Gen II ELISA (Beckman Coulter, Brea, USA) was used.

Finally, in our study DHEAS correlated negatively with age, According to some data, DHEAS concentration declines with age, although conflicting data also exists [28–30].

In conclusion, this study provides robust evidence that DHEAS is not helpful in predicting clinical pregnancy in women with have normal ovarian reserve who are undergoing first ICSI.

We could also speculate that, although that DHEAS is a metabolite of DHEA, and there is rational basis for giving DHEA supplementation to women with low ovarian reserves to improve embryo quality, there does not appear to be a rationale for such treatment in women with normal ovarian reserves [31]. Further studies are obviously needed in this field.
